# Opinions of Portuguese Veterinarians on Telemedicine—A Policy Delphi Study

**DOI:** 10.3389/fvets.2020.00549

**Published:** 2020-08-21

**Authors:** Manuel Magalhães-Sant'Ana, Maria Conceição Peleteiro, George Stilwell

**Affiliations:** ^1^Ordem dos Médicos Veterinários, Lisbon, Portugal; ^2^CIISA—Centro de Investigação Interdisciplinar em Sanidade Animal, Faculdade de Medicina Veterinária, Universidade de Lisboa, Lisbon, Portugal

**Keywords:** telemedicine, teleconsultation, code of conduct, teleconsulting, teleadvice, Portugal, telehealth, Policy Delphi

## Abstract

Telemedicine has only received limited attention by veterinary professional regulatory bodies, particularly in Europe. In Portugal, telemedicine is currently outside what is considered acceptable practice by the regulator, the Portuguese Veterinary Order (Ordem dos Médicos Veterinários). As part of a wider research aimed at gathering evidence for developing a new veterinary Code of Professional Conduct, this study describes the use of the Policy Delphi technique to gather the views and perceptions of a purposeful sample of 41 Portuguese veterinarians regarding telemedicine. Four main issues were addressed using mixed research methods: teleconsultation, teleconsulting, teleadvice, and the regulator's role. Responses highlight participants' perception of both the relevance of medical digital technologies in improving healthcare and their limitations. Overall opinion was that, although restrictions to remote veterinary practice should be reduced, improved guidance and regulation are warranted. Eighty percent of participants considered that limits to the use of veterinary telemedicine should be imposed and two thirds considered that a remote consultation must always be preceded by a face-to-face consultation. While most respondents thought that vet-to-vet teleconsulting using social media (namely Facebook) should not be banned, 83% recognized that it should be regulated by ethical standards. Participants' concerns with telemedicine had mostly to do with reputational risk for the veterinary profession, while overlooking privacy or confidentiality issues. A consultative group should be established to ensure that telemedicine providers comply with professional requirements. It is expected that these results will support policy-making by the Portuguese Veterinary Order and by veterinary regulators at other jurisdictions.

## Introduction

Telehealth (the remote exchange of health information through technological platforms) and telemedicine (the use of telehealth for diagnosis and treatment of patients) have so far received limited attention by veterinary professional regulatory bodies. The human medical profession has long embraced telemedicine as part of its armory to improve healthcare services, but its use in veterinary medicine has remained marginal, especially in Europe, where telemedicine remains mostly unregulated. The Federation of Veterinarians of Europe (FVE) has prepared a position paper and recommendations on the use of telemedicine, to be adopted by November 2020, and focused on four domains: remote consulting, remote diagnosis, remote prescribing, and third party generated medical data (1). Similar to the position of the American Veterinary Medical Association (AVMA) (2), the FVE recommends its members to allow the use of telemedicine in the context of a veterinarian-client-patient relationship (VCPR).

The benefits and barriers of telemedicine have been identified before (3–5). Benefits can include more affordable services, convenience and practicality, less distress for the animal, improved access to specialist care and more efficient triage. Possible barriers comprise the increased risk of medical error, fraud, miscommunication, and lower standards of practice. In addition, the risks of breaching the rules laid down by the General Data Protection Regulation (GDPR) should not be neglected.

In Portugal, telehealth and telemedicine currently fall outside of what is considered acceptable practice by the profession regulator, the Portuguese Veterinary Order (Ordem dos Médicos Veterinários, OMV). Provisions regarding telemedicine are absent from the OMV statutes (Law 125/2015, of Sept 3rd), and remote consultations and prescriptions are prohibited by its Code of Professional Conduct (6). Despite these restrictions, some forms of veterinary telehealth have been developed in recent years. In effect, out of a population of 6,562 active veterinarians (as for April 2020) registered with the OMV, 3,872 are members of a private Facebook group called *Fórum Veterinário de Portugal* [Portuguese Veterinary Forum]. Access to the group is controlled by a moderator, relying on the veterinary license number and the name of the Alma Mater. This dedicated social media forum has been used for almost a decade by a large proportion of the Portuguese veterinary community as a telemedicine consulting (teleconsulting) platform, where daily clinical cases are remotely shared between veterinary practitioners, especially in small animal practice. Furthermore, within the last few years, several companies have introduced innovative business models by offering remote veterinary advice (teleadvice) to pet owners through online chat (e.g., petappoint.com), video calls (e.g., veton.pt), or telephone calls (e.g., Linha Saúde Animal 24), but success has so far been limited. These telehealth services provide animal owners with first line general health advice and, if needed, refer cases to local veterinary clinics. Concerns about the legality of such services have reached the OMV Ethics Council and sparked debate about the acceptable limits of veterinary telehealth.

The COVID-19 pandemic has accelerated the worldwide implementation of telemedicine, and veterinary medicine has been no exception. The U.S. Food and Drug Administration has eased telemedicine requirements during the public health crisis (7) and the UK's Royal College of Veterinary Surgeons Council has temporarily allowed remote prescribing of veterinary medicines when no other option is available (8). The French government has just recently issued a decree establishing an 18 months experimental trial on the use of veterinary telemedicine (9). In Portugal, as soon as the state of emergency was declared (March 18, 2020), the OMV Ethics Council temporarily permitted remote consultations and prescriptions in the presence of a VCPR, and similar decisions are known to have been taken by regulators at other European countries.

Since 2018, the OMV has been preparing a new Code of Professional Conduct using an evidence-based approach, including a Policy Delphi study. The Delphi method is a well-established group facilitation technique, combining both qualitative and quantitative research methods, that enables a group of experts (usually between 20 and 50) to explore complex or contentious issues in a state of quasi-anonymity (10). The Policy Delphi is a form of Delphi study that provides the range of opinions about a given topic without having to reach a final consensus (11). With this study, we aim to describe the use of the Policy Delphi technique in late 2018 to gather the views and perceptions of Portuguese veterinarians regarding telemedicine, and thus support regulation and policy-making by the OMV and by veterinary representative organizations at other jurisdictions.

## Method

A web-based three round Policy Delphi study was held between Sept. and Dec. 2018, aiming to explore prominent topics that required improved regulation (namely telemedicine, animal welfare, and advertising) and thus gather evidence for developing a new OMV Code of Professional Conduct. The Delphi procedures will be detailed in an ensuing paper on animal welfare and are only summarized herein. Research topics emerged from a retrospective investigation of disciplinary complaints against veterinarians in Portugal (12). A purposeful sample of veterinarians that reflected the diversity of the veterinary profession in Portugal was identified using snowball sampling and 70 invitations were sent. Variables included gender, age, education, experience with veterinary policy-making, field of work, and geographical distribution.

The Policy Delphi used the platform SurveyMonkey and relied on pre-validated methodologies (13). Each round was piloted by five senior veterinarians with mixed professional backgrounds and not involved with the study. With regard to telemedicine, the study started with an introduction to the research topic, including definitions, and links for further reading were provided (Supplementary Data, in Portuguese). Participants were asked about their views on the role of the OMV in regulating telemedicine, on vet-to-vet teleconsulting and on teleadvice to animal owners using a 5-point Likert scale (1—strongly disagree; 5—strongly agree). To provide context, participants were invited to justify at least three of their answers. Following the same approach, participants were then asked about their views on teleconsultations, including remote diagnosis and prescription. Finally, building on these results, participants were invited to freely explore the research topic. A N/A option was always available.

Regarding quantitative data, Microsoft Excel was used for descriptive statistical analysis and for generating the graphics. For the qualitative data, content analysis was performed by MM-S using NVivo software, following the preparation, organizing, and resulting phases suggested by Elo and Kyngäs (14). A preliminary list of codes was created, and then refined and expanded after subsequent coding runs. The coding process was discussed with co-authors and revised appropriately. Copies of the coding matrix are available upon request. Quotes were translated to English by MM-S and amended to facilitate readership without changing their original meaning.

This study is part of a wider research project conforming to an Ethical Review Form (Ethics Council, Ordem dos Médicos Veterinários, reference number: 673/CPD/2017). Participants were invited by email and informed about the aims of the study, data storage and anonymity before data collection, and consent was granted by submitting their demographic profile. After acceptance, a code was given to each participant and their identities remained anonymous. The contact details of the researcher responsible were provided and participants could withdraw from the study at any time.

## Results

### Study Population

**Figure 1:**
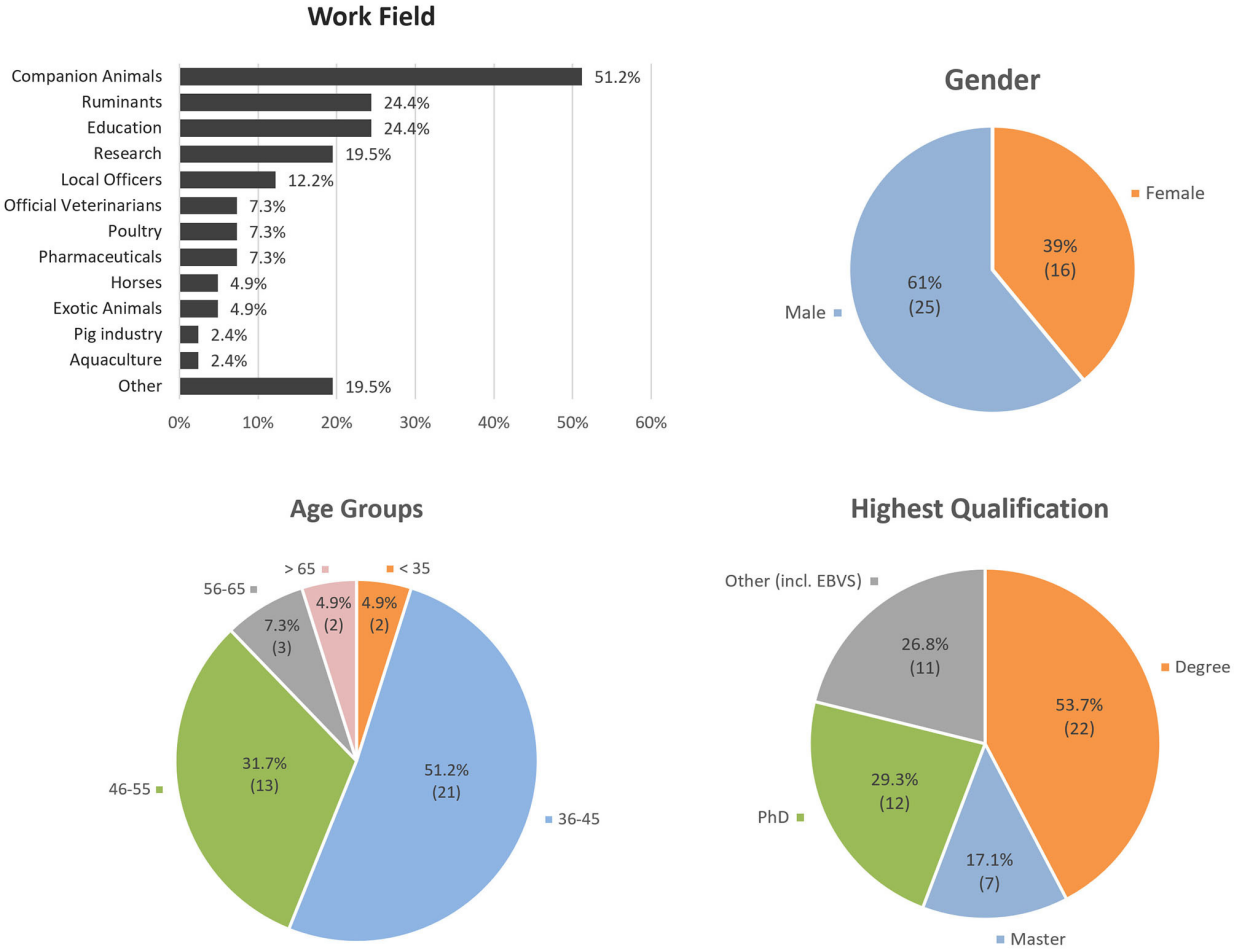
Demographic profile of veterinary participants: Work field, age groups, highest qualification, and gender. Twenty participants (49%) indicated more than one work field. “Other” fields of work included non-conventional therapies, bullfighting, nutrition, reproduction, and animal welfare. “Other Highest Qualification” also included European Board of Veterinary Specialization (EBVS^®^) Veterinary Specialists.

Forty-one veterinarians accepted to participate (59% acceptance rate), and no participant withdrew from the study (100% response rate). Their demographic profile can be found in Figure 1 and Supplementary Figure 1. In summary, participants were deemed to reflect the breadth of the veterinary profession in Portugal in terms of gender (61% Male), age (56% younger than 45), education (Degree, Master, Ph.D.), expertise (different backgrounds, including EBVS experts), field of activity (all main activities represented, with 51% working with companion animals) (Figure 1).

Thirty-four participants (84%) declared having policy-making experience at veterinary level. In terms of geographical distribution, all districts were represented, except two, and 65% of participants developed their professional activity in the six most populated districts: Lisboa (30%), Porto (18.3%), Braga (5%), Setúbal (3.3%), Aveiro (6.7%), and Faro (1.7%) (Supplementary Figure 1).

### Role of the OMV in Telemedicine

**Figure 2:**
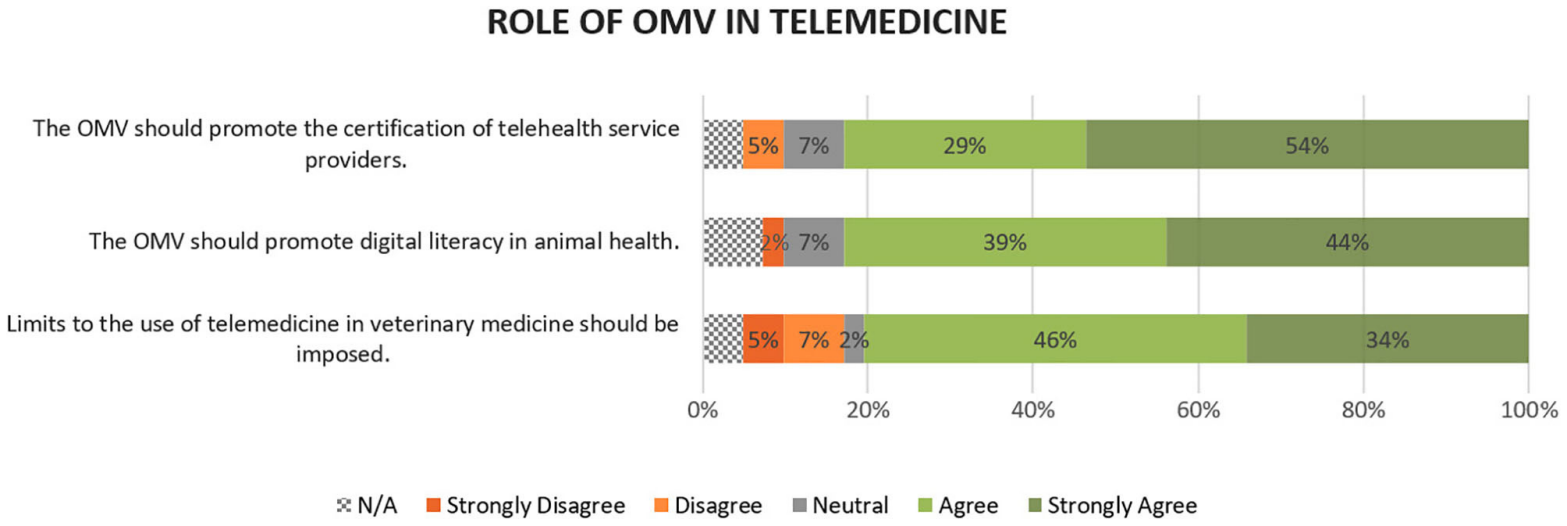
Participants' opinions on the role of OMV in regulating telemedicine. Values were rounded to no decimals.

Thirty-three participants (80%) broadly agreed (i.e., aggregate agree and strongly agree responses) that limits to the use of telemedicine in veterinary medicine should be imposed (mean ±*SD*; 4.03 ± 1.25). A clear majority (34 participants, 83%) considered that the OMV should promote both digital literacy in animal health (4.32 ± 1.26) and the certification of telehealth service providers (4.38 ± 1.09). Detailed results can be found in Figure 2. Reasons for imposing restrictions to the use of telemedicine involve ensuring quality of services and preventing fraud and misuse of information. It was suggested that the OMV should establish a permanent consultative group on veterinary telehealth, responsible for monitoring technological innovations that may have an impact in the provision of veterinary services and for issuing guidelines on telemedicine. One senior academic cautioned: “*OMV must respond in a concerted, coherent, and articulated manner, safeguarding its members and the provision of veterinary care for the general population. It must avoid case-by-case and reactive responses because they risk being ineffective and counterproductive.”* Despite these results, one small animal practitioner questioned the regulatory powers of OMV, for considering that they would create an additional obstacle to entrepreneurship and innovation.

### Teleconsultations

Twenty-eight participants (68%) broadly agreed that a remote consultation must always be preceded by a face-to-face consultation (3.82 ± 1.10). Twenty-five participants (61%) thought that remote consultations are an opportunity for improving animal healthcare (3.49 ± 1.19). Twenty-five participants (61%) broadly disagreed that remote consultations should be restricted to veterinary specialists (2.29 ± 1.11). Twenty-four (59%) also disagreed that remote consultations can endanger the reputation of the veterinary profession (2.51 ± 1.19).

**Figure 3:**
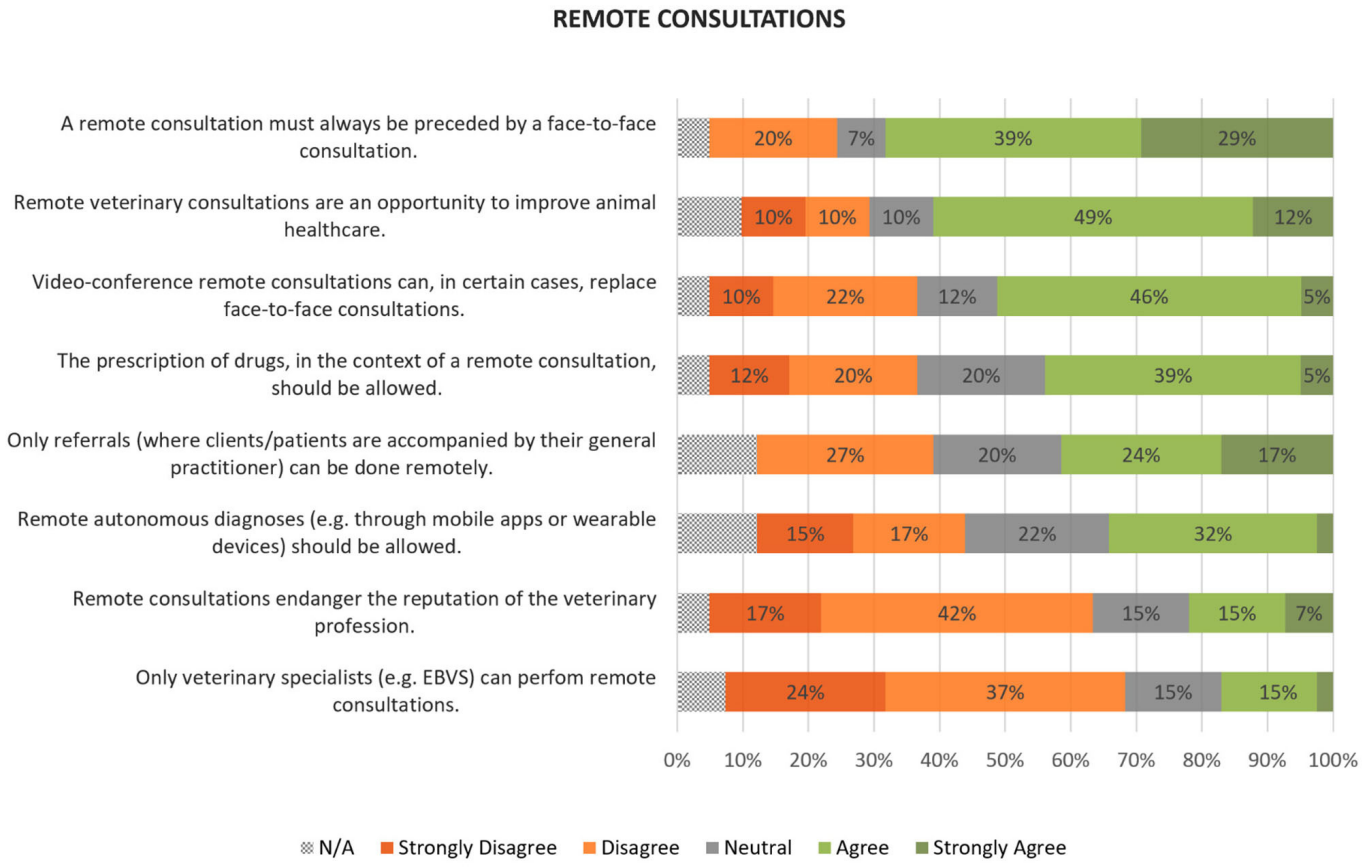
Participants' opinions on remote consultations. Values were rounded to no decimals.

A wider range of opinions was found when considering the specifics of a remote consultation. Twenty-one respondents (51%) thought that, in certain cases, video-consultations can replace face-to-face consultations but 13 (32%) disagreed (3.15 ± 1.16). Whereas, 18 (44%) agreed with remote prescription of drugs, eight (20%) were unsure and 13 (32%) disagreed (3.05 ± 1.17). A split was also found when considering whether only referrals (referral consultations in which the animals and their owners are accompanied by their general practitioner) can be done remotely (3.36 ± 1.13) and whether remote autonomous diagnoses (e.g., through mobile apps or wearable devices) should be allowed (2.89 ± 1.17). Overall results can be found in Figure 3.

In their comments, most participants acknowledged that the service provided by teleconsultations is complementary to that of physical consultations but stressed the need for having a face-to-face interaction before resorting to telematic means. Others questioned this view; a small animal practitioner noted that in urgent cases, such as poisoning and heat stroke, performing a teleconsultation “*can mean the difference between life and death*.” It was also mentioned, namely by a specialist respondent, that, in the case of behavioral medicine, since examining animals in their home environment is particularly beneficial, remote consultations should be allowed for both first consultations and follow-ups, according to the judgment of the specialist veterinarian. The prescription of medicines was only briefly mentioned. Opinions were divided between those who think that drugs should never be prescribed remotely and those who think that a drug should only be remotely prescribed if that drug had been previously prescribed for the same condition during a physical consultation. A large animal practitioner noted how animal production has been using telemedicine for years and that telemedicine “*was not born with the internet and social networks; they just gave it visibility and permanence*.”

The question of disciplinary responsibility arose, especially in the case of referral teleconsultations and how that responsibility should be shared (or not) between the referring and the referral veterinarian. Some comments denoted skepticism toward remote consultations and their benefits. Remote consultations were said to “*subvert the principles of veterinary practice*.” One local veterinary officer cautioned: “*Clinical examination of the animal is essential in 99% of cases. Unlike humans, the animal is unable to describe its symptoms and animal owners are unable to interpret them*.”

### Teleconsulting and Teleadvice

**Figure 4:**
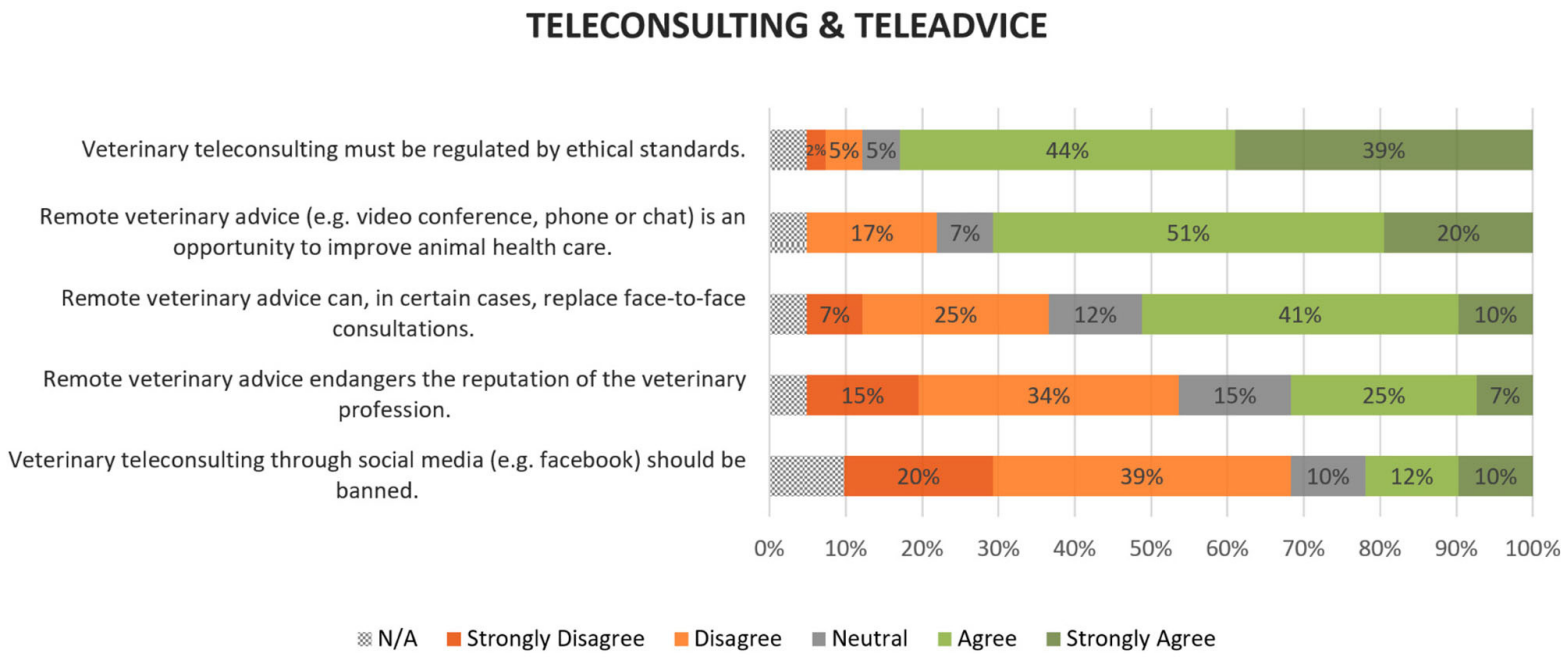
Participants' opinions on teleconsulting between veterinarians and on teleadvice to animal owners. Values were rounded to no decimals.

Thirty-four participants (83%) considered that teleconsulting between a veterinarian a veterinary consultant should be regulated by ethical standards (4.18 ± 0.99). Twenty-four (59%) broadly disagreed that teleconsulting using social media should be banned but nine (22%) broadly agreed (2.49 ± 1.28). Regarding teleadvice (between a client and a veterinary consultant), opinions were more divided: 21 (51%) were of the opinion that, in certain cases, teleadvice can replace face-to-face consultations whereas 13 (32%) broadly disagreed (3.23 ± 1.18). Twenty participants (49%) did not think that teleadvice could endanger the reputation of the veterinary profession whereas 13 (32%) thought that it could (2.74 ± 1.23). Even so, 29 participants (71%) considered that remote veterinary advice is an opportunity for improving animal healthcare (3.77 ± 0.94). Overall results can be found in Figure 4.

Regarding written comments, many participants mentioned that vet-to-vet teleconsulting, be it through social media or tele-conference, is an invaluable tool for veterinarians who are inexperienced, who work alone or in isolated regions to acquire up-to-date scientific information. One respondent compared it to attending a webinar or a conference. Teleconsulting can promote collaborative veterinary practice to fight against animal diseases and increase the range of diagnostic tools made available to animals, thus promoting animal health and welfare. The case of diagnostic imaging was underlined. Acceptable limits for vet-to-vet teleconsulting should be sought, namely through dedicated platforms, a register of competent teleconsultants and well-defined rules, and it was suggested that the OMV should bear that responsibility.

The analysis also revealed concerns about social media teleconsulting. A farm animal practitioner suggested that teleconsulting should only be carried on controlled platforms, specifically developed for that purpose, and restricted to veterinarians. Participants who agreed in banning social media teleconsulting stressed that some of the shared clinical cases, if known by the general public, could damage the reputation of the veterinary profession. A mixed (small and farm animal) practitioner noticed that “*there are colleagues who, based on the questions raised, reveal their unpreparedness for the services they apparently provide and should be considered unfit to practice (e.g., colleagues who do not work with horses, and who ask on social media which vaccines they should use, are not prepared to carry out this kind of act.)*”

With regard to teleadvice to clients, commercial telehealth service providers should be certified and must ensure that the animal is referred to a veterinary practice in case of need. Remote advice in non-urgent and distant cases would justify replacing a physical consultation. A participant with previous teleadvice experience mentioned that while some face-to-face consultations can be replaced by teleadvice (which can be considered undesirable by skeptic veterinarians), others can be anticipated or even promoted (with obvious advantages for both animals and veterinarians). Another mixed animal practitioner emphasized how small animal veterinarians have been offering teleadvice to clients by telephone and emails and how teleadvice is used routinely by farm animal practitioners and is part of modern animal farming.

## Discussion

This paper aimed at gathering the views and perceptions of Portuguese veterinarians regarding the regulation and practice of veterinary telemedicine. To the best of our knowledge, this study provides one of the first empirical investigations into the opinions of veterinary professionals on the use of telemedicine anywhere in Europe. It relied upon a Policy Delphi methodology and gathered a cohort of 41 veterinarians, representing the diversity of the Portuguese veterinary profession in terms of gender, age, expertise, area of activity, and geographical distribution. According to the 2018 FVE Survey of the veterinary profession in Europe (15), 61% of veterinarians in Portugal are up to the age of 40 and most (76%) work in small animal practice. Also, according to the FVE survey, 70% of Portuguese veterinarians are female, although the prevalence of women is much more pronounced in those under 35, a group underrepresented in our sample. The need for recruiting participants with a high level of expertise (Ph.D., specialists) and with policy-making experience excluded many younger veterinarians, and thus more female vets.

Veterinarians in Portugal have been relying on telehealth in a number of ways, thus imposing a regulatory change that current frameworks are yet to incorporate. Overall results show that regulatory restrictions to the remote practicing of veterinary medicine in Portugal should be reduced, while improving education and guidance on telemedicine. Results highlight the role of veterinary regulators in ensuring that telemedicine conforms to ethical and technical standards. The 2019 Edition of the FVE European Veterinary Code of Conduct recommends that “*Veterinarians should utilize digital and emerging technologies to enhance their provision of services as long as they can use these technologies competently, and hold up-to-date knowledge of the animal(s), of the owner and/or of the farm(s)/farmer(s)*.”[(16), p. 14]. Updating the OMV Statutes and Veterinary Code of Conduct in order to accommodate provisions on telemedicine is therefore of the upmost importance, and similar steps should be taken by veterinary regulators elsewhere.

Results convey respondents' perceptions of both the relevance of medical digital technologies in improving animal healthcare and the limitations of such technologies. In particular, participants' opinions regarding teleconsultations reflect doubts and concerns that need to be addressed. Notably, only a small majority (51%) considered that, in some cases, video-consultations can replace face-to-face consultations. Likewise, a UK online surveying of 1,230 veterinary professionals found an even split between those who thought that the Royal College of Veterinary Surgeons Code of Professional Conduct should allow for remote examination to replace physical examination in some circumstances, and those who did not (3). Results also indicate that in Portugal, at least for the study population, holding a face-to-face consultation is a pre-requisite for using telemedicine. This is in line with the position taken by other veterinary regulators that remote consultations should only be allowed within the context of a veterinarian-client-patient relationship (VCPR). The question remains on how to ensure whether a VCPR has been established or if exceptions, such as the case of behavioral medicine, should be permitted. Moreover, the concept of VCPR can have different interpretations at different jurisdictions [as illustrated by the ongoing the debate in the UK on what is meant by “under care”(4)] and any meaningful discussion on what should be considered a VCPR for the Portuguese veterinary profession requires an investigation of its own.

Remote referrals and vet-to-vet advice (herein referred as teleconsulting) are well-established telemedicine services in some countries, such as the US and the UK. In Portugal, however, dedicated teleconsulting services have had limited implementation and several reasons may be partly responsible. One reason involves the small number of veterinary specialists working in Portugal (33, according to the EBVS website). Another possible reason is that general practitioners are enjoying free consulting advice from colleagues using social media (namely through Facebook's *Fórum Veterinário de Portugal*). While most respondents thought that teleconsulting using social media should not be banned, the vast majority recognized that it should be regulated by ethical standards. One possible solution could involve licensing veterinarians, specialists or not, who may wish to perform tele-consulting. This approach is already being used in France, where veterinary tele-consultants must be licensed to practice telemedicine (9).

Participants pointed out that this form of social media interaction between veterinarians may help younger or inexperienced practitioners in dealing with their everyday clinical cases, and thus presenting an alternative to traditional means of collecting scientific information. Yet, a word of caution is in order. Since the soundness of the scientific advice currently being offered has never been investigated, nor the decision process of practitioners when faced with opposing views, there is not enough evidence to sustain the claim that social media teleconsulting promotes evidence-based veterinary medicine. Furthermore, the mechanisms in place for registering with the *Fórum Veterinário de Portugal* are not enough to ensure that all those registered are competent to provide teleconsulting or that they are, in effect, licensed veterinarians.

Significantly, concerns with teleconsulting using social media had mostly to do with reputational risk for the veterinary profession, and did not specifically address privacy or confidentiality issues. This result is even more relevant given that the breach of Facebook users' data by Cambridge Analytica—a scandal with huge ethical repercussions, namely for the veterinary community (17)—had been disclosed a few months before the Delphi was conducted. It is therefore likely that Portuguese veterinarians have little concern with Facebook's data management policies or with the thought that posting patients' clinical data on Facebook may constitute a disclosure of confidential information, potentially in breach of client confidentiality (18). Likewise, no reference was made to the GDPR and on how veterinary telemedicine can conflict with its requirements. This is however a topic that requires further scrutiny, especially since the implementation of the GDPR in veterinary practice has given rise to some confusion (19).

The disciplinary repercussions of remote teleconsulting also need further reflection. The disciplinary responsibilities of teleconsultant veterinarians need to be clarified; it seems strange that, in Portugal, a veterinarian may be accountable for transmitting incorrect or misleading advice to a client, even if by social media, but that such mechanism is not in place for vet-to-vet teleconsulting. The situation is increasingly problematic if non-EU veterinarians (not covered by the professional qualifications Directive) are involved (20). Concerns such as these have been by put forward by the OMV Ethics Council (21) but remain largely unresolved, and need to be explicitly set out in the revised Code of Professional Conduct. One possible solution could involve creating an alternative teleconsulting platform, supervised by the OMV, where veterinarians can register as certified consultants.

Veterinary teleadvice has also been looked with suspicion by the Portuguese veterinary community. The first Portuguese teleadvice platform (Linha Saúde Animal 24, a telephone line), operated for <2 years, after failing to reach economic viability, and the veton.pt platform was also discontinued. Remote advice to clients is not deemed to be considered a remote consultation provided that diagnoses, treatments, or prescriptions of drugs are not performed. However, the dividing line between a clinical advice and an actual consultation is not always clear, and the risk that illegal veterinary acts may be carried out remotely is tangible. Veterinary regulators should act to ensure that telehealth service providers comply with professional requirements, namely on informed consent, data protection, client confidentiality, and quality assurance. The suggestion of establishing a permanent consultative group on veterinary telehealth would be a step on that direction.

The use of autonomous diagnostic technologies in animals received mixed opinions which require further examination, namely by distinguishing between companion and farm animal practice since results suggest that the farm animal sector may have been more proactive in embracing telemedicine. In effect, telemedicine has already been part of modern animal farming for decades, including wearable devices to remotely detect heat or diseases, such as mastitis and lameness (22).

Telemedicine has turned into a vital healthcare tool in the post-Covid-19 world (23). Veterinary telemedicine has arguably been more developed in North America than in Europe, although in a recent survey of 76 US veterinarians only 13 said to utilize telemedicine often or fairly often (24). In turn, European veterinarians are skeptical that telemedicine will facilitate the provision and access to veterinary services in the future (15). The FVE has only recently specifically addressed telemedicine but it remains responsibility of each member state to define the rules to which telemedicine should comply. In this regard, the initiative of the French authorities to hold an 18-months experimental telemedicine trial is laudable. The experiment is open to all veterinarians, registered with the Ordre National des Vétérinaires (National Veterinary Order), who may wish to practice telemedicine and a national register will be created. A veterinary teleconsultation can only be carried out if the animal has been subjected to a physical consultation within the last 12 months by the same veterinarian or by a veterinarian practicing within the same veterinary practice (9). The decision of the OMV to permit teleconsultations within the context of a VCPR during the COVID-19 outbreak needs to be followed by structured measures that may ensure the traceability, quality, and ethical responsibility of telemedicine services in the future.

This study used the Policy Delphi technique and mixed research methods. The generated opinions gave an in-depth view on teleconsultation, teleconsulting, teleadvice, and on the role of the OMV in regulating telemedicine, but several issues remained unanswered. Results regarding the remote prescription of medicines are insufficient to draw meaningful conclusions and the risks of telemedicine on antimicrobial resistance and on animal welfare should be further investigated. Although results from this study cannot be directly translated into the overall Portuguese veterinary population, it is expected that they may reflect the range of opinions of the target population. However, since the emergence of the COVID-19 pandemic and the recent widespread use of veterinary telemedicine, the views of Portuguese veterinarians is likely to have evolved. Further research on this topic in therefore required.

## Data Availability Statement

The raw data supporting the conclusions of this article will be made available by the authors, without undue reservation.

## Ethics Statement

The studies involving human participants were reviewed and approved by Conselho Profissional e Deontológico, Ordem dos Medicos Veterinários. The patients/participants provided their written informed consent to participate in this study.

## Author Contributions

MM-S designed and conducted the study, performed formal analysis, and prepared the original draft manuscript. MP and GS supervised the study and validated the analysis. All authors reviewed and edited the manuscript.

## Conflict of Interest

MM-S and MP are members of the OMV Ethics Council since 2016 but were not involved in the ethical approval of the research project. MM-S is member of the FVE Statutory bodies Working Group, and has contributed to the FVE telemedicine position statement. The remaining author declares that the research was conducted in the absence of any commercial or financial relationships that could be construed as a potential conflict of interest.
