# Supplementary material for: Opinions of Portuguese Veterinarians on Telemedicine—A Policy Delphi Study
Source: Front Vet Sci. 2020 Aug 21;7:549. doi: 10.3389/fvets.2020.00549 (PMC7472629; doi:10.3389/fvets.2020.00549)
Supplement: Supplementary file 1 [file Data_Sheet_1.PDF]

Policy Delphi - Round 1 – Information for Participants regarding Telemedicine

A telemedicina - a troca de informações médicas sobre um paciente através de plataformas tecnológicas - é hoje uma realidade na medicina veterinária em Portugal. Empresas como a vet.on e a Petappoint oferecem aconselhamento veterinário gratuito à distância de um clique, através de video chamada e chat (mensagem instantânea). Ambas as plataformas afirmam não ter por objetivo dar consultas remotas (i.e. à distância) mas sim fornecer informações e tirar dúvidas aos tutores de animais. Recentemente, a Linha Saúde Animal 24 - a primeira linha telefónica nacional de aconselhamento veterinário, lançada em Dezembro de 2016 - encerrou por insustentabilidade económica.

Por outro lado, é cada vez mais comuns aos médicos veterinários recorrerem às redes sociais para partilharem casos clínicos e pedir o conselho remoto de colegas sobre diagnósticos e tratamentos (o designado *teleconsulting*). Aproveitando esta tendência, existem empresas de consultoria veterinária prontas a entrar no mercado do *teleconsulting* (que é uma prática comum em países como o Reino Unido), o que deu origem a um parecer por parte da OMV.

Embora inovadores, estes serviços não representam ainda uma mudança de paradigma em que verdadeiras consultas remotas (principalmente através de video-conferência) convivem com consultas presenciais, o que já acontece em algumas especialidades da medicina humana, como é o caso da cardiologia pediátrica. Mais uma vez, por não ser considerada uma profissão da área da saúde, a medicina veterinária nao está integrada no Sistema Nacional de Avaliação de Tecnologias de Saúde (SiNATS) nem na Comissão de Avaliação de Tecnologias de Saúde (CATS).

Por fim, é também importante considerar o advento dos chamados *wearable devices*, dispositivos vestíveis, que permitirão monitorizar pacientes durante 24 horas (e.g. medidor de glicémia em pacientes diabéticos), realizar diagnósticos remotos em tempo real (e.g. hipoglicémia) e mesmo tratamentos (e.g. injeção de insulina), tudo sem a intervenção directa de um médico veterinário.

Para consultar o parecer da OMV relativas às plataformas on-line de consultoria, clique aqui.

Para se familiarizar com a terminologia na TeleSaúde, clique aqui.

Para ler uma reportagem sobre TeleSaúde humana, clique aqui.
Para consultar o Código Deontológico, clique aqui.
