# Supplementary material for: Opinions of Portuguese Veterinarians on Telemedicine—A Policy Delphi Study
Source: Front Vet Sci. 2020 Aug 21;7:549. doi: 10.3389/fvets.2020.00549 (PMC7472629; doi:10.3389/fvets.2020.00549)
Supplement: Supplementary file 2 [file Image_1.PDF]

Supplementary Material

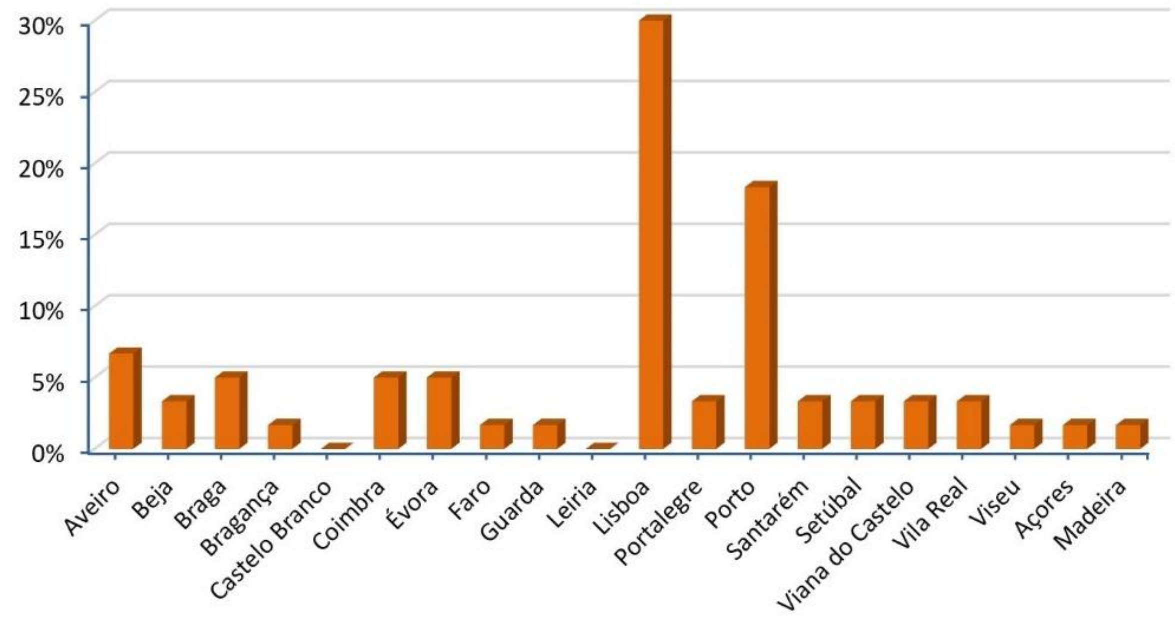

Supplementary Figure 1. Geographical distribution in terms of professional activity. Ten participants (32%) selected two or more districts/regions (n = 60).
